# Investigation of the effectiveness of parent-child interaction therapy on adjustment and behavioral problems in children with subthreshold autism

**DOI:** 10.3389/fpsyg.2024.1408847

**Published:** 2025-01-22

**Authors:** İsmail Seçer, Sümeyye Ulaş, Eda Tatlı, Fatmanur Çimen, Burcu Bülbül, Beyzanur Tosunoğlu

**Affiliations:** ^1^Atatürk University, Department of Guidance and Counseling, Erzurum, Türkey; ^2^Erzurum Technical University, Department Psychology, Erzurum, Türkey

**Keywords:** ASD, subthreshold ASD, PCIT, intervention, school refusal, parents

## Abstract

**Background:**

Autism Spectrum Disorder (ASD) is an increasingly prevalent neurodevelopmental disorder. However, the number of children who exhibit subthreshold levels of ASD symptoms, significant enough to experience functional and adaptive difficulties, is also substantial. These children require early interventions, similar to those diagnosed with ASD, due to their exhibited adaptive and behavioral challenges. PCIT offers a unique opportunity for children and their parents exhibiting subthreshold ASD to address such challenges through its practices and techniques.

**Aim:**

This study aims to assess the initial result effectiveness of standard PCIT on the adaptive skills and school refusal behaviors of children exhibiting subthreshold ASD, as well as on the levels of parental stress and caregiving burden. The study intends to test these effects and report the outcomes.

**Method:**

This study is a case study, and it involves three children with subthreshold ASD symptoms and their parents. The therapy processes were conducted for approximately 1 year.

**Result:**

Results obtained from this study revealed that PCIT holds promising prospects for improving adaptive and interaction skills, reducing school refusal, and alleviating parental stress and caregiving burden among children exhibiting subthreshold ASD.

**Discussion:**

PCIT is considered a functional approach for children and parents demonstrating subthreshold ASD symptoms, besides interventions addressing diagnosed ASD children. It is suggested that future studies should evaluate the effectiveness and sustainability of PCIT through randomized controlled trials in the context of subthreshold ASD.

## Introduction

Autism Spectrum Disorder (ASD), characterized by impairments in social interaction and communication, restricted interests, and repetitive behaviors, is a common neurodevelopmental disorder (Kamio et al., 2013; Ma et al., 2022). As reported in the 2023 report of the Autism and Developmental Disabilities Surveillance (ADDM) Network of the Centers for Disease Control and Prevention (CDC), approximately 1 in every 36 children around the age of 8 may have ASD (Kamio et al., 2013). In DSM-4, Asperger’s Syndrome and Autism were considered separate disorders. However, in DSM-5, they have been merged as different severity levels of the same disorder spectrum (Sanders, 2009). Consequently, many cases previously attributed to Asperger’s Syndrome are now evaluated under ASD with DSM-5. Furthermore, the similarities between many diagnostic criteria in the social communication disorder category and ASD present a challenge for clinicians in making accurate diagnoses (Dell'Osso et al., 2016a). Another difficulty in diagnosing ASD is the presence of children with subthreshold ASD, whose symptoms closely resemble those of children diagnosed with ASD but are milder in terms of social-cognitive and emotional aspects (Dell’Osso et al., 2015; Gökçen et al., 2014). Increasing literature emphasizes the need to consider subthreshold autistic traits from a psychopathological perspective (Dell'Osso et al., 2015, 2016a, 2016b; Donati et al., 2019) and indicates that the distribution of these individuals within the general population shows significant variations (Hoekstra et al., 2007; Ronald and Hoekstra, 2011; Skuse et al., 2005). Symptoms observed in subthreshold cases bear significant resemblance to those of diagnosed ASD children, albeit with milder social-cognitive and emotional symptoms compared to ASD (Dell’Osso et al., 2015; Gökçen et al., 2014). Studies have reported *limited friendship relationships, low personal* and *social adaptive skills*, and various emotional problems among children with subthreshold ASD (Jobe and White, 2007; Kanne et al., 2009; Kamio et al., 2013). These and similar findings regarding subthreshold autism cases led researchers to propose the *Subthreshold Autism Spectrum Model* (Donati et al., 2019).

Dell'Osso et al. (2016a) suggested that autism, as conceptualized in the DSM-5 (Diagnostic and Statistical Manual of Mental Disorders, Fifth Edition), should not only refer to the core features of ASD within the clinical population but also acknowledge the dimensional nature of these features and the continuity between clinical and subclinical presentations. Moreover, it was suggested that the “spectrum” concept should include not only core ASD symptoms but also mild/atypical symptoms, gender-specific qualities, behavioral indicators, and personality traits associated with ASD. Dell'Osso et al. (2016b) proposed that the Subthreshold Autism Spectrum has seven domains, including *childhood and adolescence* (excessive silence, avoidance of eating and social interactions), *non-verbal communication* (avoidance of eye contact and touch), *verbal communication* (peculiar and monotonous speech), *low empathy* (difficulty understanding others’ thoughts, facial expressions, and intentions), *inflexibility and adherence to routines* (difficulty in changing daily habits), *restricted interests and rumination* (excessive interest in certain topics, numbers, etc.), and *limited response to emotional stimuli* (unresponsiveness to pain, temperature, or sounds; Donati et al., 2019; Dell’Osso et al., 2019). Researchers suggested that many symptoms exhibited in these seven different domains by children with subthreshold ASD might not be adequately recognized by clinicians and mental health professionals, potentially leading to strong associations with different disorders in adolescence and adulthood (Dell’Osso et al., 2018; Kamio et al., 2013; Takara and Kondo, 2014). Since underlying ASD symptoms are not adequately addressed, symptoms may remain misunderstood, and it leads to increased treatment resistance and chronicity (Donati et al., 2019; Kamio et al., 2013; Shirama et al., 2022). It was noted that this situation may cause various problems in adult life, including severe psychiatric disorders (Dell’Osso et al., 2018; Kato et al., 2013). Data reported in the literature indicates a strong relationship between ASD and subthreshold cases and a range of mental health issues, many of which are comorbid with other disorders (Dell’Osso et al., 2019; Mito et al., 2014; Takara and Kondo, 2014). The comprehensive psychiatric and developmental assessment and treatment of this population, often diagnosed and treated late, have significant implications, necessitating further in-depth studies (Kamio et al., 2013).

Evidence-based interventions for children with ASD have become increasingly widespread in recent years (Nevill et al., 2018; Green et al., 2010; Althoff et al., 2019; Pickles et al., 2016; Green et al., 2018; Green et al., 2022; Allen et al., 2023; Ke et al., 2017; Moody and Laugeson, 2020; Masse et al., 2016; McInnis et al., 2020; Owen et al., 2020; Scudder et al., 2019; Vetter, 2018; Tobin et al., 2014; Wong et al., 2015; Wolstencroft et al., 2018). Parent-mediated interventions offer significant advantages as they reduce the demands on children with ASD compared to traditional behavioral approaches, bringing treatment into home and community settings and facilitating the transfer of skills to real-life situations (Conrad et al., 2021). In addition, since parents already spend a lot of time with their children, these interventions provide a cost-effective opportunity with extensive implementation and generalization in everyday life and across different contexts (Shalev et al., 2020). Approaches focusing on early childhood education have been reported to have a noticeable impact on the social communication and interaction skills, as well as the quality of life, of children with ASD (Green et al., 2010; Pickles et al., 2016; Owen et al., 2020; Tobin et al., 2014; Ke et al., 2017).). However, it was emphasized that involving parents as a significant part of the intervention process in early childhood interventions for children with ASD is crucial both for ensuring the sustainability of the intervention’s effects and for transferring them to different contexts (Dai et al., 2018; Turgeon et al., 2021). Furthermore, it was reported that the active involvement of parents in the intervention process can lead to significant gains for themselves and improvements in areas such as parental stress and emotional regulation skills (McInnis et al., 2020; Masse et al., 2016; McNeil et al., 2018; Pan et al., 2023; Ulaş, 2022). Therefore, early parent-based interventions are considered vital for children with ASD and subthreshold symptoms (Brookman-Frazee et al., 2006; McConachie and Diggle, 2007).

In recent years, within evidence-based practices for intervening with children with ASD, Parent–Child Interaction Therapy (PCIT) emerged as a significant approach (McInnis et al., 2020; Masse et al., 2016; Quetsch et al., 2022; Pacia et al., 2021). PCIT, developed by Sheila Eyberg for the rehabilitation of children aged 2–8 with emotional and behavioral problems, is an evidence-based intervention approach that references play therapy, behavior therapy, and attachment theory (Eyberg and Funderbuck, 2011). In PCIT, while parent–child interaction is strengthened in line with attachment theory, positive behavioral changes in children are addressed via behavioral theory and methods (Niec, 2018). PCIT consists of two standard phases following detailed teaching sessions for parents (Eyberg and Funderbuck, 2011; Masse et al., 2016). In the first phase, which is called Child-Directed Interaction (CDI), parent–child interaction is enhanced, aiming to help parents utilize basic skills referred to as PRIDE skills (Praise, Reflection, Imitation, Description, Enjoy) at an expert level, while also teaching them to avoid negative skills such as criticism, questioning, and commanding (Eyberg et al., 2014; Masse et al., 2016). The second phase, Parent-Directed Interaction (PDI), focuses on strengthening the child’s compliance skills. In this phase, parents’ effective command-giving and follow-through skills are developed through simultaneous coaching. The therapy process is successfully concluded when the expertise criteria outlined in the PCIT therapy protocol are met for both phases (Eyberg and Funderbuck, 2011; Eyberg et al., 2014; Masse et al., 2016).

The California Evidence-Based Clearing House for Child Welfare (California Evidence-Based Clearinghouse for Child Welfare, 2021) identifies Parent–Child Interaction Therapy (PCIT) as the “gold standard” in evidence-based practices and parent education for rehabilitating problem behaviors observed in children. Most therapies provided to families of children with ASD involve intensive interventions directed by therapists. However, Parent–Child Interaction Therapy (PCIT) is a cost-effective, time-limited intervention designed to help parents address behavioral issues. PCIT may serve as a transitional therapy to more intensive treatments and can be used as a primary treatment to prepare children with ASD for other comprehensive therapies (McNeil et al., 2018).

In recent years, researchers have increasingly focused on Parent–Child Interaction Therapy (PCIT) for children diagnosed with or showing symptoms of ASD. Initial findings indicate that PCIT is effective in various behavioral areas, including disruptive behavior, adaptive functioning, atypical behaviors, prosocial verbalizations, social awareness, and child compliance (Agazzi et al., 2013; Armstrong et al., 2015; Lesack et al., 2014; Masse et al., 2016; Zlomke et al., 2017). However, there are significant differences in the results of efficacy studies of PCIT for children diagnosed with ASD or exhibiting high-functioning characteristics within this population.

Current research consistently reports that PCIT is an effective approach for reducing disruptive behaviors and improving adaptive and parenting skills in children diagnosed with ASD or those with high-functioning ASD (Cambric and Agazzi, 2019; Vess and Campbell, 2022; Scudder et al., 2019; Han et al., 2021; Masse et al., 2016; McInnis et al., 2020; Zlomke and Jeter, 2020; Allen et al., 2023).

However, there are significant differences in the research findings regarding the effectiveness of PCIT in reducing or improving autism-related symptoms in children with ASD. Vess and Campbell (2022) reported significant improvements in ASD symptomatology with PCIT. Ginn et al. (2017) found improvements in social awareness, Zlomke et al. (2017) noted enhancements in functional communication skills and prosocial behaviors, and Parladé et al. (2020) reported significant effects and improvements in social awareness, social responsiveness, and reductions in restrictive/repetitive behaviors with PCIT.

However, some research indicates that PCIT is not effective in reducing ASD symptoms (Scudder et al., 2019; Zlomke and Jeter, 2020), improving receptive language skills (Allen et al., 2023), or enhancing social skills such as imitative play, social participation, and eye contact (Lieneman et al., 2019). These differences in research findings are thought to be due to variations in research designs and the developmental characteristics of the children with ASD studied. Therefore, more evidence and efficacy trials are needed to better understand the effectiveness of PCIT in this population.

Another observed difference in research on PCIT interventions for children with ASD relates to parental stress. Scudder et al. (2019) reported that PCIT is not effective in reducing parenting stress in parents of children with ASD. In contrast, Ginn et al. (2017), Allen et al. (2023), and McInnis et al. (2020) found that PCIT is effective in reducing parenting stress, and Agazzi et al. (2017) reported that it is effective in reducing symptoms of anxiety and depression in parents.

Despite the differences in research findings, it is believed that PCIT is a promising approach for children with ASD or at risk of ASD, as well as their parents, and that further clinical investigation is needed. Based on this need, the present study expands the research on the effectiveness of PCIT by including children with subthreshold ASD symptoms, who represent a group at risk for an ASD diagnosis, and their parents in the PCIT intervention.

In this study, the effect of PCIT on school refusal behavior in children with subthreshold ASD symptoms was also examined. The aim was to contribute to expanding the scope of PCIT’s effectiveness in the context of school attendance issues. School refusal is defined as a phenomenon that includes severe symptoms like complete or partial absenteeism, chronically being late for school, developing deliberate behavior attempting to skip school in the morning, or accelerating the demand for future absence (Kearney and Bensaheb, 2006). School refusal is a problematic behavior that manifests with the child’s unwillingness to stay at school due to the strong negative emotions he/she feels at school and the desire not to come to school. It is also suggested that school refusal, which is considered an increasingly common condition in child psychiatry, should be considered as a child mental health problem (Blumkin, 2016; Kearney and Albano, 2004). Frequent absence from school increases the risk of low academic achievement, risk behaviors, substance use, and mental health problems (Epstein et al., 2020; Gottfried, 2009). Munkhaugen et al. (2017) noted that children with ASD have lower social motivation, exhibit more introverted and depressive symptoms, and face significant issues with school refusal, highlighting the need for specialized interventions for these children. Although the literature on school refusal in children with ASD is limited, it is reported that school refusal behavior in these children ranges between 40 and 53%, which is considerably higher compared to typically developing children (5–28%; Havik et al., 2015; Munkhaugen et al., 2017; Nordin et al., 2024). Adams (2022) found that school absenteeism in children with ASD is three times higher than in typically developing children, with school refusal being the most prominent reason, and that parental mental health issues are strongly related to school refusal in these children. Therefore, PCIT can be considered a promising early intervention program for children with ASD who exhibit school refusal and their parents. In their meta-analysis, Nordin et al. (2024) reported that despite the higher prevalence of school attendance issues among children with ASD compared to their typically developing peers, prevention and intervention approaches in this area are quite limited. Education is defined by UNESCO (2020) as a fundamental human right that prepares children and young people for life and the future. Thus, early interventions are essential for children with ASD who have three times more school attendance issues than their peers. Based on this necessity, this study specifically examined the effectiveness of PCIT on school refusal behavior in children with subthreshold ASD symptoms and aimed to contribute to the expansion of the literature in this direction.

The primary aim of this research is to evaluate the effectiveness of standard PCIT in reducing emotional and behavioral problems and school refusal behavior in children with subthreshold ASD symptoms, as well as in alleviating parenting stress and caregiver burdens in their parents, through a case series involving three children.

In addition to quantitative measurements, participants’ views have been analyzed to establish social validity evidence and comprehensively evaluate the effectiveness of PCIT. In this context, the hypotheses and questions provided below were addressed during the research process:

**H1**: Standard PCIT is an effective intervention approach in reducing adjustment and behavioral problems in children with subthreshold ASD.

**H2**: Standard PCIT is an effective intervention approach in reducing school refusal in children with subthreshold ASD.

**H3**: Standard PCIT is an effective intervention approach in reducing parental stress and caregiving burden.

## Participants and characteristics

The research was conducted with three different children with subthreshold ASD and their parents. The primary inclusion criteria were having a diagnosis of subthreshold ASD and exhibiting school refusal behavior. Consequently, reports from the Child Psychiatry Clinic at Erzurum City Hospital and assessments and observations conducted by the researchers were decisive in the selection process. Following comprehensive psychiatric evaluations at the Child Psychiatry Clinic of Erzurum City Hospital, six children diagnosed with subthreshold ASD were referred to the PCIT clinic at Atatürk University. These children and their parents were invited to the clinic, where their Eyberg Child Behavior Inventory (ECBI), ASD, school refusal, parenting stress, and caregiver burden scores were determined. Only three children met the inclusion criteria for the PCIT intervention. A key inclusion criterion was that each case had disruptive behavior and adjustment issues, as evidenced by scores of at least 114 on the Intensity subscale and at least 17 on the Problem subscale of the ECBI. Moreover, a score higher than 50% of the maximum score on the school refusal scale, parenting stress scale, and caregiver burden measurements was essential. However, Case 1 had an ECBI Intensity score of 96, which is below the criterion value of 114, and a Problem score of 16, one point below the criterion value of 17. Despite this, it was deemed appropriate to include Case 1 in the therapy process due to the high observed ASD symptoms, school refusal scores, and the parent’s stress and caregiver burden scores, as well as observations made by the researchers.

Moreover, the presence of a mental disorder that would prevent the child or parent from participating in the therapy process, being involved in a different therapy or intervention, etc., were used as exclusion criteria. For parents, exclusion criteria included having a psychotic disorder, undergoing psychiatric treatment, continuing with a different therapy or parenting program, or having a condition that would prevent regular participation in the therapy process. Of the three children admitted to PCIT, both parents of two children and only the mother of one child (Case 1) were involved in the therapy process. Descriptive and demographic information about the cases is presented below.

Case 1: Kürşat Kaan: He is 4 years old and the only child of his parents. His mother is a teacher, and his father is a police officer. Kaan, noticed by his parents to exhibit limitations in communication and interaction since the age of 2, was sent to preschool for the development of his social skills. However, due to the pandemic, he had to take a break for about a year.

Despite starting kindergarten after the pandemic, the child exhibits intense crying and resistance to separation from their parents almost every morning. There is significant school refusal symptoms reported by both the parents and teachers. Even though he has developed speech skills, there is an accompanying intense echolalia. Kaan, who is inadequate in functional play, shows excessive interest only in the spinning wheels of cars. He prefers spending time and playing with his father more but only chooses cars in his play with his father. During moments of joy, he exhibits stereotypical behaviors obsessively, which greatly disturbs the family. Concurrent with the therapy process, Kürşat Kaan also started attending preschool. His parents expressed that he isolates himself in the class and has difficulties interacting with his peers, and they attribute this to the pandemic’s effect of excessive solitary confinement at home. They also mentioned facing significant resistance when initially sending him to preschool in the mornings. As a result of a psychiatric examination, it was found that Kürşat Kaan significantly meets the symptoms of ASD, but a subthreshold diagnosis was made, recommending monitoring for a while and receiving special education. In line with this recommendation, he started receiving 2 h of special education per week. During the intake assessment, it was determined that Kürşat Kaan has good speech skills but speaks with a mixed sentence structure, lacks adequate eye contact, dislikes initiating contact, is good at focusing on and sustaining play, and is generally calm. The parents, on the other hand, were found to be quite unhappy, in a state of search, and stressed. They expressed that they expected the therapy to reduce stereotypical behaviors, improve communication skills, make him able to socialize and integrate into school and peer groups, and strengthen interactions between them, mentioning that they do not have any training in this field.

Case 2 Mert: He is 4 years old and the younger one of two siblings. His father works as an engineer and his mother works as a sales manager. Following the emergence of behavioral issues related ASD in Mert, his mother left her job. Despite not fully meeting the criteria for autism spectrum disorder (ASD) on repeated psychiatric evaluations, Mert received a subthreshold ASD diagnosis. He has been attending daycare since the age of 1.5 and lacks any close friends. He has not yet completed toilet training. Mert exhibits an excessive and repetitive interest in the letter “P” and the color pink. While capable of drawing and coloring, he consistently prefers similar drawings and colors (especially the letter “P”). Descriptions from parents and daycare teachers characterize him as having weak impulse control, difficulty following instructions, appearing inattentive when spoken to, and being quick to tears. He also shows weak interest in peers and his siblings, and experiences difficulty in verbal communication and forming sentences. During speech, Mert refers to himself as “he” or “him.” For example, “He came,” “He did,” etc. Furthermore, he remains passive in social settings requiring interaction (such as school, park, etc.). Interaction with his mother is better than with his father and he can engage in prolonged play sessions. It was noted that he struggles with mutual conversation, expressing himself, defining emotions, exhibits pronounced obsessive behaviors, and has limited eye contact. In terms of school refusal, it was determined that the child exhibited behaviors such as not wanting to go to school in the mornings, crying when getting on the school bus, and preferring to stay unhappy and alone during the first hours at school. Furthermore, during assessment interviews, both parents exhibited signs of psychological fatigue, depressive symptoms, and high levels of stress.

Case 3 Görkem: He also is 4 years old and the only child of his parents. Both parents work in healthcare, but his mother left her job after Görkem’s diagnostic process. Görkem is described as a child who struggles to communicate in his social life, easily distracted, impulsive, and unable to sit still due to his hyperactivity. Moreover, he exhibits significant echolalia. With limited eye contact, Görkem refers to himself as someone else. Besides finding joy in scattering toys around and displaying aggressive behaviors towards his mother, his developmental history reveals discrepancies between the use of meaningful words at 1.5 years old and the lack of coherence between actions and their conceptual meanings (“take the car for me” instead of “give me the car”). Milestones such as walking, and toilet training were achieved within the expected time frame. His parents characterized him as hyperactive, having insufficient attention span, impulsivity, lack of forethought before action, prone to anger outbursts, easily angered, unresponsive when spoken to, difficulty following instructions, inability to foresee danger, quick to tears, excessively anxious, experiencing eating habit issues, and becoming upset when unable to find his way. Following psychiatric evaluation, he was recommended for special education support under the diagnosis of ASD, and a monitoring period was agreed upon for further diagnosis. Information obtained from Görkem’s parents and teachers indicates that they have significant difficulty getting him ready for and sending him to school in the mornings. During school hours, he prefers to be alone, shows reluctance to participate in social activities and make friends, and exhibits crying behavior during the first hours of separation from his parents.

### Therapy process

This research, being a case study, was conducted according to the ABA design. The ABA design is a single-case design that includes three phases. In this context, A represents the baseline level before the intervention, B represents the intervention implemented to achieve the expected change, and A again represents the return to the baseline level before the intervention, meaning the withdrawal of B (Johnson and Christensen, 2024). Within this framework, for each of the three children studied, measurements of ECBI and school refusal were taken before the intervention, along with measurements of parenting stress and caregiver burden from the parents. Following this, the standard two-phase PCIT (Parent–Child Interaction Therapy) intervention was applied. The intervention was withdrawn once the parents met the mastery criteria for PCIT.

According to the DPICS-4 coding system used in the PCIT process (Eyberg et al., 2014), for parents to reach the mastery criteria in the CDI stage, they are expected to use each of the categories label praise, behavior description, and reflection at least 10 times in 5 min, while a total of 0 use is expected from the categories question, command, and negative talk. For parents who reach this condition and move on to the PDI stage, in addition to maintaining the CDI mastery criteria, the total number of effective commands in the five-minute PDI coding process should be 75%, the ratio of effective commands to effective follow-through should be at least 75%, the ability to follow time-out procedures correctly if used, and the ECBI intensity score should fall below 114 (Eyberg and Funderbuck, 2011).

The therapy process was completed face-to-face with two cases and online with one case. The reason for conducting one case online was the family residing at a location that is significantly distant from the clinic, as well as the impossibility of providing face-to-face services. For this case, teaching sessions and therapy sessions were conducted via the Zoom platform, and sessions were recorded with parental consent. For the other two cases, the processes were conducted in the PCIT therapy room within the university premises, and similarly, all sessions were recorded. In cases 2 and 3, both mothers and fathers actively participated in therapy, whereas only the mother was involved in the process for case 1. The therapy processes also encompassed a part of the training for researchers 1 and 2 under the PCIT within Agency Training program and the cases that researchers 4, 5, and 6 needed to work on as part of their PCIT therapist training. Due to the inability to initiate therapy processes simultaneously, all therapy practices for each case lasted between 3 to 6 months, and all sessions for the three cases were completed within approximately 1 year.

Case 1 took a long time due to the difficulties associated with online treatment and was completed in 17 sessions. In Case 2, both parents attended the sessions, and the process was completed in 11 sessions. Case 3 was completed in a total of 15 sessions and was affected by some family problems experienced by the family.

After completing the therapy process for each case, families and children were invited to the clinic for a special session 3 months later to conduct follow-up measurements. During this session, the parents’ PRIDE skills were observed, and the children’s ECBI and school refusal scores were measured.

## Measures

To determine the effectiveness of the applied intervention, reliability, and validity have been assessed using the following measurement tools, which have been internationally recognized and validated within Turkish culture.

Eyberg Child Behavior Inventory (ECBI): The Eyberg Child Behavior Inventory (ECBI) is a 36-item caregiver-report measure for children aged 2–16 years (Eyberg and Pincus, 1999). The ECBI is composed of two subscales: Intensity and Problem. Caregivers rate the intensity of their child’s disruptive behaviors on a 7-point Likert-type rating system and indicate whether each behavior is a problem for them on a dichotomous “yes” or “no” scale. To ensure linguistic equivalence and cultural appropriateness, Seçer and Ulaş (2022) undertook an adaptation of the ECBI to the Turkish context, using data obtained from 812 caregivers. To assess the fit of the scale, confirmatory factor analysis was conducted, yielding satisfactory model fit indices (*χ*^2^/df: 1.2, RMSEA: 0.063, RMR: 0.041, NFI: 0.96, NNFI: 0.96, CFI: 0.97, GFI: 0.95, AGFI: 0.86), indicating a good level of fit. Although CFA was conducted for the Turkish sample, no norms were established. For this reason, the standards of other countries (for example, Taiwanese) that have similarities with Turkish culture in terms of child behavior and parenting approaches are taken as a basis. Accordingly, the EBCI density cutoff score for Taiwanese boys aged 2–6 is 131 and 124 and 124 and 118 for girls, respectively. The problem cut-off score was 17 for boys and 13 for girls (Chen et al., 2018).

The Turkish version of the Dyadic Parent–Child Interaction Coding System-IV (DPICS-IV; Eyberg et al., 2013). The Dyadic Parent–Child Interaction Coding System—Fourth Edition (DPICS; Eyberg et al., 2014) is an observational measure of caregiver and child behaviors. Caregivers are observed with their children during brief play interactions. For the current study, caregiver verbalization codes and child compliance codes were utilized. See Table 1 for definitions of each code. By explicitly coding observational data, caregivers’ verbal or physical behaviors are evaluated. This observational data is important in terms of the examining parallels between changes in parenting skills and changes in child behavior.

**Table 1:** Characteristics of children administered with PCIT at the beginning of therapy.

Parameter	Characteristic	*n*	%
Age	2–4	1	33.3
	4–6	2	66.6
Who the child lives with	Both parents	2	66.6
	One of parents	1	33.3
Migration background	Yes	1	33.3
	No	2	66.6
Preschool/Kindergarten attendance	Normal preschool	1	33.3
	Special care preschool	1	33.3
	Kindergarten/Daycare	1	33.3
Psychiatric diagnosis	Subthreshold ASD	3	100
Intellectual disability	Average	3	100
	Below Average	0	0
	Mild Intellectual Disability	0	0

School Refusal Assessment Scale-Parent Form: Initially developed by Kearney and Silverman (1996), later revised by Heyne et al. (2017), and subsequently adapted to Turkish culture by Seçer (2014), serves as an instrument to evaluate school refusal behaviors exhibited by children and adolescents. The 24-item measure with a four-factor structure was validated within the Turkish cultural context, demonstrating favorable fit indices (*χ*^2^/df = 2.21, RMSEA = 0.061, NFI = 0.97, CFI = 0.98, GFI = 0.94). For this study, scale items were modified to assess for school refusal tendencies observed by caregivers in children aged 2 to 7 years. Consequently, confirmatory factor analysis was conducted based on data collected from 738 caregivers, confirming that 18 items of the scale exhibited satisfactory fit (*χ*^2^/df = 3.62, RMSEA = 0.06, NFI = 0.96, CFI = 0.97, GFI = 0.93). For this form, Cronbach’s alpha was found to be.884 and the standard deviation was 5.51.

Parenting Stress Scale: The Parenting Stress Index (PSI), originally developed by Abidin (1982) and subsequently adapted to Turkish culture by Mert et al. (2008), aims to assess stress arising from caregiver-child interactions. This 36-item scale utilizes a 5-point Likert scale and encompasses three factors. The internal consistency coefficients were 0.81 for the parental distress subscale, 0.78 for the difficult child subscale, 0.76 for the parent–child dysfunctional interaction subscale, and 0.71 for the total parental stress index score. The standard deviation was 17.6.

Zarit Caregiving Burden Scale: The Zarit Caregiver Burden Scale was developed by Zarit et al. (1980) and adapted into Turkish by İnci and Erdem (2006) and Özlü et al. (2009). The scale is a Likert-type measurement tool consisting of 22 items. The responses are scored from “0, never” to “4, almost always.” The minimum possible score on the scale is 0, and the maximum is 88, with higher scores indicating a higher level of caregiver burden. During the adaptation process, the internal consistency coefficient of the scale was found to be 0.83. Arıkan (2020) found the internal consistency coefficient to be 0.96. The psychometric properties of the scale were re-examined by the researchers and an internal consistency coefficient of 0.86 was calculated. In addition, it was determined that the model fit for construct validity was at a good level (*χ*^2^/df: 2.3, RMSEA: 0.073, RMR: 0.067, NFI: 0.91, NNFI: 0.91, CFI: 0.90).

Interview form: It was used to evaluate the therapy process, and the changes observed in the child after the successful completion of the therapy process with the parents during the research process. The interview questions were prepared by the researchers. It includes questions used in a face-to-face interview immediately after the graduation session to learn the evaluations of the parents and to explain the quantitative findings. Examples of questions are as follows; How do you interpret the change that participation in the PCIT process has created in you and your child? What is the most important change that PCIT has created in you as a parent?

### Data analysis

To clinically test the changes in ECBI, school refusal, parental stress, and caregiver burden measurements from baseline through treatment stages and post-treatment, the Reliable Change Index (RCI) was used. The RCI is a statistically based analysis technique used to determine whether changes (increases or decreases in scores) in a case’s measurement scores are genuine or due to measurement error, thereby assessing clinical significance. Clinical prediction systems, which can go beyond traditional statistical methodologies (such as pretest-posttest differences, mean scores, etc.), should accompany the reporting and evaluation of results (Cañete-Massé et al., 2018). The RCI has been reported to perform similarly to more complex regression formulas (Heaton et al., 2001; Temkin et al., 1999). It helps clinicians ascertain whether the change resulting from the intervention reflects random chance, measurement error, or the effects of the intervention itself (Tröster et al., 2007).

An RCI value equal to or greater than 1.96 is considered statistically significant (at the 95% confidence interval) from pre-to post-treatment (Jacobson and Truax, 1991). An RCI value less than −1.96 indicates a clinically significant decrease, while a value higher than 1.96 indicates a clinically significant increase. In RCI calculations, findings from the Turkish standardization study were used, resulting in values of 0.92 for the ECBI scale (Seçer and Ulaş, 2022), 0.88 for the school refusal scale (Seçer and Ulaş, 2020), 0.88 for parental stress (Mert et al., 2007), and 0.83 for caregiving burden (Özlü et al., 2009). Cohen’s d statistic was calculated to determine effect sizes. Cohen’s d value, a measure of effect size for the magnitude of change in scores, was examined. A Cohen’s d effect size value less than 0.20 indicates no effect; between 0.20 and 0.50 indicates a small effect; between 0.50 and 0.80 indicates a medium effect; and greater than 0.80 indicates a large effect (Cohen, 1998). In case studies, the criteria for interpreting the Cohen’s d value change. Accordingly, if it is less than 0.87, it is considered a small effect size, if it is between 0.87 and 2.67, it is considered a medium effect size, and if it is above 2.67, it is considered a large effect size (Parker and Vannest, 2009).

Finally, percentage change (post-treatment score—baseline score)/baseline score) × 100 was used to report changes observed in treatment.

## Results

Findings regarding the hypotheses tested for children and parents showing subthreshold ASD are presented below in order.

The first hypothesis of the study, “*PCIT is an effective intervention approach in reducing adjustment and behavioral problems in children with subthreshold ASD*,” was tested using the results obtained from pre-treatment, treatment stages, and follow-up measurements from the ECBI intensity and problem scales, and the results are presented in Table 2.

**Table 2:** ECBI Intensity and problem measurements in children with subthreshold ASD.

Participants	ECBI Intensity					ECBI Problem				
	Mean	SD	Cohen’s d	Perc. change (%)	RCI	Mean	SD	Cohen’s d	Perc. change (%)	RCI
Case 1 Kağan Mother										
Baseline										
CDI pre-post test	87.25	6.14	−1.26	−8	1.50	13.50	2.12	−1.18	−16	1.68
PDI pre-post test	58.2	13.19	−1.73	−28	**3.97**	6.00	3.13	−1.60	−45	**2.70**
Base-PDI post-test	73.5	18.04	−1.19	−54	**5.25**	10.00	4.78	−1.25	−81	**4.48**
Follow			1.41	16	0.75		1.41	1.41	67	−0.67
Case 2 Mert Mother										
Baseline										
CDI pre-post test	125.67	8.98	−1.37	−9	1.93	16.50	1.11	−1.35	−8	0.67
PDI pre-post test	88.40	15.85	−1.99	−26	**4.94**	9.40	3.83	−0.94	−28	**3.71**
Base-PDI post-test	111.17	22.05	−1.22	−46	**6.58**	13.67	4.24	−1.02	−72	**4.48**
Follow		4.95	1.41	9	−0.75		0	-	0	−1.01
Case 2 Mert Father										
Baseline										
CDI pre-post test	120.33	13.52	−1.23	−12	**3.00**	13.33	4.42	−1.51	−33	**3.37**
PDI pre-post test	84.60	16.62	−1.47	−22	**4.72**	5.83	1.83	−1.18	−27	**2.02**
Base-PDI post-test	106.83	23.78	−1.27	−53	**7.41**	10.58	5.12	−1.84	−75	**5.17**
Follow		7.07	1.41	15	−1.07		0.71	1.41	20	−0.67
Case 3 Görkem Mother										
Baseline										
CDI pre-post test	110.86	22.61	−1.47	23	**7.19**	10.86	5.08	−1.60	−43	**4.04**
PDI pre-post test	57.80	9.23	−2.08	25	**2.15**	3.20	1.05	−1.72	−36	1.01
Base-PDI post-test	93.00	33.87	−1.51	−60	**8.95**	8.54	5.19	−2.01	−79	**5.17**
Follow		2.12	1.41	5	−0.32		0	-	0	0.00
Case 3 Görkem Father										
Baseline										
CDI pre-post test	116.86	20.04	−1.50	−21	**4.51**	12.57	5.26	−1.41	−37	**4.38**
PDI pre-post test	87.00	16.43	−1.10	−17	**3.54**	3.8	2.43	−1.32	−46	**2.02**
Base-PDI post-test	107.69	24.78	−1.59	−51	**7.72**	9.77	5.92	−1.73	−90	**6.20**
Follow		5.66	1.41	11	0.85		1.41	1.41	100	−0.67

^*^Bold values > 1.96, *p* < 0.001.

Given the results obtained from the RCI analysis, there was no statistically significant difference in the intensity scores of ECBI (Eyberg Child Behavior Inventory) exhibited by children showing subthreshold ASD (Autism Spectrum Disorder) in the CDI (Child-Directed Interaction) phase between the mother of Case 1 and the mother of Case 2; however, there was a significant difference between the father of Case 2 and the measurements of both the mother and father of Case 2, with RCI values exceeding 1.96 (*p* < 0.001). In the PDI (Parent-Directed Interaction) phase, it was determined that there was significant variation in the ECBI intensity scores of all cases. The findings indicate significant changes in problematic behaviors of children exhibiting subthreshold ASD as the intervention progresses and as treatment is maintained across different stages. Examination of Cohen’s d effect size values confirms the results of the RCI analysis.

Upon examining the RCI analysis results for ECBI problem measurements, in the CDI phase, no significant change was found in the ECBI Problem scores of the mothers of Case 1 and Case 2 (*p* > 0.001); however, there was significant differentiation in the measurements of the father of Case 2 and the parents of Case 3, with RCI values exceeding 1.96 (*p* < 0.001). In the PDI phase, it was observed that the RCI values for the mother of Case 3 were not significant (*p* > 0.001); however, the RCI values for the mothers of Case 1 and Case 2, and the fathers of Case 2 and Case 3 exceeded 1.96, indicating significant differentiation. Examination of Cohen’s d effect size values confirm the results of the RCI analysis and suggests that PCIT (Parent–Child Interaction Therapy) is an effective approach in reducing ECBI intensity and problem scores in children with subthreshold levels of ASD. In addition, it was determined that the maintenance test results did not show a significant difference, and the effectiveness obtained from the post-test score was preserved in the measurements made 3 months after the intervention.

The second hypothesis of the study, “*PCIT is an effective intervention approach in reducing school refusal in children with subthreshold levels of ASD*,” was tested by the results obtained from the Parent Form of the School Refusal Assessment Scale during pre-treatment, treatment stages, and follow-up measurements, and the results are presented in Table 3.

**Table 3:** School refusal results of children with subthreshold ASD.

Participants	Mean	SD	Cohen’s d	Percent change (%)	RCI
Case 1 Kağan Mother					
Baseline					
CDI pre-post test	35.75	2.88	−1.48	−11	1.22
PDI pre-post test	29.80	2.16	−1.48	−10	1.04
Base-PDI post-test	33.2	4.26	−1.59	−33	**2.26**
Follow		2.83	1.41	15	−0.69
Case 2 Mert Mother					
Baseline					
CDI pre-post test	35.67	5.23	−1.79	−21	**2.26**
PDI pre-post test	26.6	4.72	−0.72	−11	**2.08**
Base-PDI post-test	31.55	6.33	−2.13	−52	**4.34**
Follow		4.24	1.41	30	−1.04
Case 2 Mert Father					
Baseline					
CDI pre-post test	46.67	4.23	−1.02	−8	1.91
PDI pre-post test	31.20	5.92	−1.49	−22	**2.61**
Base-PDI post-test	39.64	9.24	−1.23	−51	**4.52**
Follow		3.54	1.41	20	−0.87
Case 3 Görkem Mother					
Baseline					
CDI pre-post test	42.71	4.17	−1.27	−11	**2.08**
PDI pre-post test	27.60	5.37	−1.57	−23	**2.43**
Base-PDI post-test	36.42	8.78	−1.32	−54	**4.52**
Follow		3.54	1.41	23	−0.87
Case 3 Görkem Father					
Baseline					
CDI pre-post test	46.86	7.20	−1.13	−15	**2.95**
PDI pre-post test	31.40	3.33	−1.98	−17	1.39
Base-PDI post-test	41.54	9.63	−1.40	−45	**4.34**
Follow		3.54	1.41	17	−0.87

^*^Bold values > 1.96.

In children exhibiting subthreshold levels of ASD, RCI analysis results indicated significant differentiation in school refusal concerning post-test scores compared to baseline scores, with RCI values exceeding 1.96 (*p* < 0.001). Moreover, PCIT emerged as an effective approach in reducing school refusal in these children. Examination of Cohen’s d effect size values confirmed the RCI analysis results, thus substantiating the research hypothesis.

The third hypothesis of the study, formulated as “*Reducing parenting stress and caregiving burden is an effective intervention approach*,” was tested using findings obtained from pre-treatment, treatment phases, and follow-up measurements, with the results presented in Table 4.

**Table 4:** Parental stress and caregiving burden in parents of children with subthreshold ASD.

Participants	Parental Stress					Caregiving Burden				
	Mean	SD	Cohen’s d	Perc. change (%)	RCI	Mean	SD	Cohen’s d	Perc. change (%)	RCI
Kağan Mother										
Baseline										
CDI pre-post test	44	5.76	−1.04	−12	1.30	36.50	4.77	−0.94	−11	1.22
PDI pre-post test	31.20	3.88	−1.75	−18	0.54	26	2.16	−1.85	−13	0.56
Base-PDI post-test	38.20	8.56	−1.38	−44	**2.38**	31.70	6.88	−1.35	−39	1.78
Follow		0	-	0	0.00		1.41	1.41	8	−0.56
Mert Mother										
Baseline										
CDI pre-post test	46.67	8.75	−1.18	−18	**2.27**	39.33	5.09	−1.70	−18	1.45
PDI pre-post test	26.40	7.01	−1.23	−25	1.73	25.60	4.88	−1.93	−27	1.45
Base-PDI post-test	37.45	12.87	−1.52	−65	**4.00**	34.33	8.81	−1.55	−54	**2.89**
Follow	28	1.41	−1.41	−7	−1.08		2.83	1.41	18	−0.45
Mert Father										
Baseline										
CDI pre-post test	48.83	7.91	−1.54	−20	**2.27**	45.50	4.31	−0.12	−1	0.89
PDI pre-post test	38.80	5.83	−0.21	−3	1.08	34.60	3.19	−1.07	−9	0.78
Base-PDI post-test	45.67	9.29	−1.65	−51	**3.35**	41.00	6.77	−0.74	−33	1.67
Follow		0.71	1.41	3	−0.11		2.83	1.41	13	−0.45
Görkem Mother										
Baseline										
CDI pre-post test	50.86	7.59	−1.34	−17	**2.27**	52.29	5.47	−1.41	−13	1.67
PDI pre-post test	38.40	4.72	−0.34	−4	0.86	35.00	8.07	−1.24	−22	**2.23**
Base-PDI post-test	46.85	9.54	−1.48	−48	**3.13**	46.23	11.09	−1.24	−58	**3.90**
Follow		2.12	1.41	9	−0.32		4.24	1.41	24	−0.67
Görkem Father										
Baseline										
CDI pre-post test	54.43	8.01	−1.32	−16	**2.59**	51.29	6.56	−1.02	−12	1.78
PDI pre-post test	38.80	5.27	−0.42	−5	1.08	32.3	6.65	−1.47	−23	**2.00**
Base-PDI post-test	49.23	11.06	−1.43	−52	**3.67**	44.46	11.77	−1.15	−59	**3.78**
Follow		2.83	1.41	13	−0.43		5.66	1.41	33	−0.89

Bold figures indicate significant values in RCI.

The RCI analysis results regarding parenting stress in parents of children with subthreshold levels of autism spectrum disorder (ASD) indicated a significant differentiation from pre-intervention scores among all parents, with RCI scores exceeding 1.96 (*p* < 0.001), demonstrating that Parent–Child Interaction Therapy (PCIT) is an effective approach in reducing parenting stress. Cohen’s d effect sizes also corroborate the findings of the RCI analysis. Furthermore, the follow-up test on RCI analysis results indicates that the effectiveness achieved through the intervention persists, with stress levels continuing to remain low 3 months post-intervention.

Regarding the RCI analysis results on caregiving burden, except for one parent (Case 2—father), significant differentiation and reduction in caregiving burden scores compared to pre-intervention were observed among all parents. Cohen’s d effect size values also support these findings. The follow-up test on RCI analysis results also indicates that the effectiveness of the intervention persists, with caregiving burden levels continuing to remain low 3 months post-intervention.

The findings obtained regarding parenting stress and caregiving burden confirm the third research hypothesis.

## Qualitative findings

Findings from pre-session and post-intervention interviews with parents indicated a noticeable increase in interaction and communication skills in their children, the establishment of a warm and secure relationship, an enhanced sense of competence and determination in disciplining their children, and a significant reduction in behavioral and emotional problems. These findings are consistent with the current literature on PCIT from various studies (Bjørseth and Wichstrøm, 2016; Eisentadt et al., 1993; McNeil and Hembree-Kigin, 2010; Nixon et al., 2004; Phillips and Mychailyszyn, 2023; Thomas et al., 2017).

The parents of Case 1 reported rapid progress in Kürşat Kağan’s speech and communication skills, increased willingness to attend school, and closer engagement with peers. Teachers also noted Kürşat Kağan exhibiting much more social behavior, actively participating in games, and abandoning isolating behaviors. The parents of Case 2 reported that their child resumed preschool attendance with the CDI phase, showed increased enthusiasm for attending, and did not encounter any complaints or disruptive behaviors related to school by the time the PDI phase was reached, indicating complete cessation of school refusal. The parents of Case 3 mentioned that they re-enrolled their child in preschool immediately after the termination of therapy due to adjustment issues, and according to supported reports from teachers, Görkem’s speech and communication skills had significantly improved, enabling better self-expression and participation in social activities, resulting in enjoyment. They also noted that Görkem no longer resisted going to school and did not exhibit any crying spells or similar behaviors.

## Discussion

In this research process, the focus has primarily been on the adaptation and interaction skills of children displaying subthreshold ASD symptoms. In this regard, scientific processes have been conducted to report the effectiveness of PCIT in a significant area of concern in the current literature. PCIT was reported as an effective approach in improving the adaptation and interaction skills of children diagnosed with ASD exhibiting atypical development (Bjørseth and Wichstrøm, 2016; Eisentadt et al., 1993; McNeil and Hembree-Kigin, 2010; Nixon et al., 2004) and in reducing parenting stress and difficulties in emotion regulation (Thomas and Zimmer-Gembeck, 2012; Timmer et al., 2005). However, no study reporting its effectiveness on children and parents displaying subthreshold symptoms could be found. Despite subthreshold ASD children exhibiting milder ASD symptoms when compared to children diagnosed with ASD, it is noted that emotional and behavioral problems can be prevalent (Dell’Osso et al., 2018; Donati et al., 2019; Kato et al., 2013; Takara, 2014).

PCIT is considered to be one of the most effective approaches in enhancing the interaction between parents and children with typical and atypical development, as well as in developing adaptive skills (Phillips and Mychailyszyn, 2023; Thomas et al., 2017; Ward et al., 2016). Accordingly, it is anticipated that CDI intervention in children displaying subthreshold ASD will strengthen interactions with parents, leading to emotional and behavioral changes similar to those in typically and atypically developing children. The PDI phase, coupled with positive parenting skills, is predicted to reduce destructive behavioral tendencies and enhance adaptive skills in children. Findings from the research process have demonstrated that PCIT is an effective approach in improving adaptive skills and reducing disruptive behaviors in children with subthreshold ASD (Ke et al., 2017; Owen et al., 2020; Tobin et al., 2014). Consequently, a significant decrease in ECBI intensity and Problem scores from pre-therapy to CDI and throughout the intervention to PDI was observed in all three cases, with this decrease being substantially maintained in follow-up measurements. The obtained results not only demonstrate the effectiveness of PCIT in reducing disruptive behaviors in children with subthreshold ASD but also are deemed to significantly contribute to addressing the limitations perceived in intervention practices for subthreshold ASD in the literature.

Another variable addressed in children with subthreshold levels of ASD during the research process is school refusal. School refusal is reported as a common problem in children with ASD (Ulaş, 2022). It is noted that children displaying subthreshold ASD exhibit significant problems in school and social life (Donati et al., 2019), and various psychiatric issues can be observed (Dell’Osso et al., 2018; Kato et al., 2012; Takara, 2014). Therefore, school refusal in children at subthreshold levels of ASD is considered a significant risk factor for prominently emerging as a common problem and triggering secondary issues. Indeed, severe levels of school refusal behavior were observed in two of the three cases studied in the research process (Görkem and Mert), while mild levels were observed in one case (Kürşat Kaan). Despite being enrolled in preschool before therapy, Cases 2 and 3 had to interrupt schooling due to severe adjustment problems. Their parents expressed inadequacy in finding solutions and adopting the correct approach in this regard.

The results obtained in this study indicate a significant decrease in school refusal scores for all three children. These findings are consistent with studies reporting the effectiveness of PCIT on school refusal (Ulaş, 2022) and anxiety-related issues (Mosayebi et al., 2021; Phillips and Mychailyszyn, 2021) among children exhibiting subthreshold ASD. Therefore, PCIT emerges as an effective approach in reducing problematic behaviors, such as school refusal, presumed to be anxiety-based, in children displaying subthreshold ASD. These findings are considered significant in contributing to the diversification of applications targeting behavioral and adjustment problems in children with subthreshold symptoms, as well as in testing the effectiveness of PCIT across different samples and problem domains.

During the research process, the effectiveness of PCIT on the parents of children displaying subthreshold ASD was also examined in various contexts since it is acknowledged that the psychological characteristics of parents significantly influence and shape their children’s social, emotional, and behavioral development (Hotchkiss and Gordon Biddle, 2009). Previous studies indicated that parents of children with special needs experience higher levels of stress, depression, anxiety, and burnout in comparison to other parents, with lower levels of psychological well-being (Duran and Barlas, 2014). Therefore, it is plausible that the negative condition these parents experience in terms of their own psychological well-being may also affect their interactions with their children. Stone et al. (2015) stated that unmanageable parenting stress gradually transforms into problematic parenting behaviors over time and is strongly associated with observed destructive behavior problems in children. Karaca and Kılıç (2021) reported that parents of children diagnosed with ASD harbor intense anxiety about their children’s future and feel inadequate in overcoming the challenges they face. Research suggests that active involvement of parents in early intervention can lead to significant gains for them, including improvements in parenting stress and emotional regulation skills (Masse et al., 2016; McNeil et al., 2018; Pan et al., 2023). In this sense, PCIT is considered one of the most effective approaches in addressing parenting stress (Coley et al., 2014; Eyberg et al., 1995; Leung et al., 2016; Lyon and Budd, 2010; Thomas and Zimmer-Gembeck, 2007; Thomas et al., 2017). However, its effectiveness on the parents of children displaying subthreshold ASD has not yet been reported. Therefore, the findings of this study could play an important role in addressing this limitation. The results indicate that although parenting stress was quite high in all parents before the intervention, it significantly decreased during the therapy process, indicating the effectiveness of the intervention.

Another important variable considered among parents is the burden of caregiving. Parents of children with special needs face challenges and stress factors in social, physical, emotional, economic, etc., domains (Sav et al., 2023; Karaca and Kılıç, 2021). Due to these challenges, it is possible for them to develop various psychological symptoms (Norlin and Broberg, 2013; Duran and Barlas, 2014; Sav et al., 2023). Therefore, it is thought that the difficulties encountered by parents of children with subthreshold ASD in their daily lives, peer relationships, and school life will challenge them in the context of caregiving burden (Duran and Barlas, 2014; Karadağ, 2009; Tayaz and Koç, 2018). The results achieved in this study revealed a significant decrease in the burden of caregiving for parents. It is thought that this decrease is a significant determinant of the improvement observed in the therapy process regarding the improvement of parent–child interaction, reduction in emotional and behavioral problems in the child, and understanding and execution of commands. In the literature, there is no study reporting an intervention aiming to reduce the caregiving burden in parents of children diagnosed with ASD or having subthreshold level ASD. Therefore, it is considered that the results of this study will play an important role in strengthening early parent-based interventions in terms of caregiving burden and will contribute to overcoming the limitations in this regard.

In conclusion, PCIT yielded comprehensive positive outcomes for children with subthreshold level ASD and their parents. While supporting children’s adaptation and interaction skills on the one hand, it also contributes to reducing parenting stress and caregiving burden on the other hand. It is thought that this contribution will have a positive impact on the quality of life of both children with subthreshold level ASD and their parents, and thus, may influence secondary positive outcomes in the short and long term.

## Limitations

It is believed that the differing research results regarding the effectiveness of PCIT (Parent–Child Interaction Therapy) on children with ASD (Autism Spectrum Disorder) and their parents should prompt researchers to consider and investigate the reasons behind these discrepancies.

Even though the present study reports significant results for the literature in the PCIT field, it also has certain limitations. One notable limitation is the pilot nature of the study, focusing on only three children with subthreshold levels of ASD and lacking a randomized design. While full fidelity to the standard PCIT protocol and regular monthly supervision processes were maintained, the limited number of participants and potential internal validity issues in the research design are acknowledged. Therefore, conducting randomized controlled trials (RCTs) of PCIT with children who exhibit subthreshold symptoms and their parents will contribute to broadening the perspective in this area. In addition, the fact that all three cases in the therapy process were male and that the age groups were close to each other is considered a significant limitation, suggesting the importance of considering gender and age factors in future research. Furthermore, the necessity to conduct therapies online with one case may also be considered a limitation. Despite these limitations, it is thought that these results contribute to expanding the PCIT’s area of use.

## Data Availability

The datasets presented in this study can be found in online repositories. The names of the repository/repositories and accession number(s) can be found in the article/supplementary material.
